# Oxidative Stress in Methylmercury-Induced Cell Toxicity

**DOI:** 10.3390/toxics6030047

**Published:** 2018-08-09

**Authors:** Alessandra Antunes dos Santos, Beatriz Ferrer, Filipe Marques Gonçalves, Aristides M. Tsatsakis, Elisavet A. Renieri, Anatoly V. Skalny, Marcelo Farina, João B. T. Rocha, Michael Aschner

**Affiliations:** 1 Department of Molecular Pharmacology, Albert Einstein College of Medicine, Bronx, NY 10461, USA; beatriz.ferrervillahoz@einstein.yu.edu (B.F.); filipe.goncalves@einstein.yu.edu (F.M.G.); 2 Laboratory of Toxicology, Medical School, University of Crete, 71003 Heraklion, Greece; aristsatsakis@gmail.com (A.M.T.); medp2011622@med.uoc.gr (E.A.R.); 3 Department of Medical Elementology, Peoples’ Friendship University of Russia (RUDN University), Moscow 150000, Russia; skalny3@microelements.ru; 4 Laboratory of Biotechnology and Applied Bioelementology, Yaroslavl State University, Yaroslavl 150014, Russia; 5 All-Russian Research Institute of Medicinal and Aromatic Plants (VILAR), Moscow 150000, Russia; 6 Department of Biochemistry, Federal University of Santa Catarina, Florianopolis 88040-900, Santa Catarina, Brazil; marcelo.farina@ufsc.br; 7 Department of Biochemistry, Federal University of Santa Maria, Santa Maria 97105-900, Rio Grande do Sul, Brazil; jbtrocha@yahoo.com.br

**Keywords:** methylmercury, oxidative stress, molecular mechanisms

## Abstract

Methylmercury (MeHg) is a hazardous environmental pollutant, which elicits significant toxicity in humans. The accumulation of MeHg through the daily consumption of large predatory fish poses potential health risks, and the central nervous system (CNS) is the primary target of toxicity. Despite well-described neurobehavioral effects (i.e., motor impairment), the mechanisms of MeHg-induced toxicity are not completely understood. However, several lines of evidence point out the oxidative stress as an important molecular mechanism in MeHg-induced intoxication. Indeed, MeHg is a soft electrophile that preferentially interacts with nucleophilic groups (mainly thiols and selenols) from proteins and low-molecular-weight molecules. Such interaction contributes to the occurrence of oxidative stress, which can produce damage by several interacting mechanisms, impairing the function of various molecules (i.e., proteins, lipids, and nucleic acids), potentially resulting in modulation of different cellular signal transduction pathways. This review summarizes the general aspects regarding the interaction between MeHg with regulators of the antioxidant response system that are rich in thiol and selenol groups such as glutathione (GSH), and the selenoenzymes thioredoxin reductase (TrxR) and glutathione peroxidase (Gpx). A particular attention is directed towards the role of the PI3K/Akt signaling pathway and the nuclear transcription factor NF-E2-related factor 2 (Nrf2) in MeHg-induced redox imbalance.

## 1. Introduction

Mercury (Hg) is a global pollutant ubiquitously present in the environment with a potential toxic effect in humans. Hg can be emitted to the atmosphere by natural sources (volcanoes and forest fire) or by anthropogenic sources (industrial activities, mining, and coal combustion), and deposits into aquatic systems, where it is primarily found [1]. The inorganic mercury in the aquatic environment can be biomethylated by aquatic sulfate-reducing bacteria forming methylmercury (MeHg) [2]. MeHg has a significant biomagnification potential and accumulates along the food chain by more than seven orders of magnitude, reaching higher concentrations in large predatory fish [3]. Therefore, fish consumption is the main source of MeHg exposure in humans [4,5]. After ingestion, MeHg is absorbed from the gastrointestinal tract (around 90–95%) and is distributed to all organs and systems, however, the central nervous system (CNS) is the most sensitive organ to MeHg-induced toxicity [6,7,8,9,10,11]. In this regard, it has been demonstrated that the developing CNS is particularly vulnerable to MeHg when compared to adults (i.e., the mature CNS) [12,13]. Several epidemiologic studies have shown that MeHg is able to produce severe cognitive deficits after prenatal and postnatal exposures [14,15,16]. Indeed, deficits in neurons and glia including abnormal migration, differentiation, and growth have been associated with prolonged pre- and/or perinatal exposure to MeHg, even at moderate doses [17,18]. In addition, studies with animals have shown the effects of developmental exposure to MeHg on animal behavior, such as reduced motor activity [19], decrease in memory [20,21,22] and learning [23], among others. Furthermore, there is evidence of a correlation between heavy metals’ (including Hg) exposure and several skeletal deformities in fish larvae [24].

In addition, behavioral deficits in locomotor activity and motor performance were demonstrated in adult mice exposed to MeHg [25,26,27]. Indeed, the symptoms of MeHg poisoning in adults are frequently associated with loss of neuronal cells in the visual cortex and the cerebellum [28]. Even though the relationship between MeHg-induced motor deficit and cerebellar damage is a well-described phenomenon [29], the cellular mechanisms mediating MeHg-induced neurotoxicity have yet to be fully understood. In this regard, it is well documented that the electrophilic abilities of MeHg allow this toxicant to bind to soft nucleophilic groups, such as thiol (-SH) and selenol groups [1,12,30], disrupting the structure and activity of a large number of proteins and lead to disruption of various intracellular functions. Furthermore, a number of mechanisms have been identified as critical factors in MeHg-induced cell damage, including induction of oxidative stress via overproduction of reactive oxygen species (ROS) or reduction of antioxidant defenses and disruption of glutamate and calcium (Ca^2+^) homeostasis [1,31,32,33]. The impairment of astrocytic glutamate transport by MeHg can lead to an overproduction of ROS, since increased glutamate concentrations in the synaptic cleft can cause hyperactivation of N-methyl D-aspartate (NMDA) type glutamate receptors, leading to an increase in intracellular Na^+^ and Ca^2+^ [34], which is associated with generation of ROS [35]. In fact, inhibition of MeHg-induced ROS production was demonstrated in neurons treated with N-methyl-D-aspartate (NMDA) receptor antagonists [36]. Moreover, decreased antioxidant enzyme activity, such as glutathione peroxidase (Gpx), thioredoxin (Trx), and thioredoxin reductase (TrxR) [1,37,38,39], has been associated with increased ROS generation after exposures to MeHg, as well as the MeHg-induced mitochondrial dysfunction. MeHg can target specific thiol-containing proteins in the mitochondria, including respiratory chain complexes, leading to mitochondrial membrane potential loss and generation of ROS [40,41].

To summarize the current state of pertinent literature: (1) thiol and selenol groups have critical roles in governing MeHg pharmacokinetics; (2) the oxidative stress has a pivotal role in mediating MeHg-induced toxicity; (3) the nuclear factor erythroid 2–related factor 2 (Nrf2) can regulate the cellular response for attenuation of oxidative stress elicited by environmental toxicants [42]; (4) the phosphatidylinositol 3 (PI3) kinase/Akt pathway is essential for cell survival and can regulate Nrf2-mediated antioxidant and detoxification reactions [43,44]; this review summarizes the current knowledge regarding the molecular mechanisms involved in MeHg-induced oxidative stress and cell toxicity. Considerable attention has been directed towards the role of thiols and selenols in MeHg-induced redox imbalance, as well as the regulation/modulation induced by this metal of the transcription factor Nrf2 and PI3K/Akt signaling pathway.

## 2. Thiols and Selenols Play Fundamental Roles in MeHg-Induced Toxicity

Hg compounds react specifically with sulfhydryls (-SH) groups of cellular proteins and nonprotein molecules, forming stable complexes with defined stoichiometry, -S-Hg-R. MeHg’s affinity for the anionic form of thiol (-SH) groups is extremely high and responsible for most of its toxicological effects [45]. Indeed, in biological media, MeHg is always complexed to -SH-containing ligands [46,47,48,49,50] and the therapeutic agents effective in reducing its body-burden contain -SH groups [51]. In this regard, it is noteworthy that the MeHg distribution to the different organs, as well as its excretion and, consequently, toxicity are likely related to its interaction with -SH groups on various biomolecules. MeHg interacts with the -SH group from non-protein molecules like glutathione (GSH), and also binds to l-cysteine forming the MeHg-l-cysteine complex, which is taken up by cells from different tissues by molecular mimicry as a surrogate of methionine [52]. Particularly important, MeHg-l-cysteine conjugates serve as a substrate for the neutral amino acid transporter, LAT-1, which transports MeHg (complexed with l-cysteine) across membranes [53]. Complexes of MeHg-cysteine and MeHg-GSH have been identified in blood [46,47], and complexes with MeHg-GSH in brain [48], liver [49], and bile [50].

Selenium (Se) is an essential trace element that is an integrant part of several enzymes (selenoenzymes) in the form of selenocysteine (Sec) [54]. Selenoenzymes play critical roles in the maintenance of cellular homeostasis since the reactivity of the selenol group (-SeH) in Sec is high [55,56]. Since selenols chemically resemble thiols (-SH) it also serves as a soft ligand for complexation with soft metals [57]. Given that under physiological conditions, selenols have a lower pKa (around 5.3) when compared to thiols, the fully ionized isoform (selenolates, -Se^−^) are predominant and also more susceptible to electrophilic reactions by mercurials [58]. Five glutathione peroxidases (GPx) and three thioredoxin reductases (TrxR) are among the well-known redox-active selenoenzymes, with a selenol group in their redox active sites [55,56].

As previously mentioned, MeHg’s affinity for sulfhydryls and selenols interferes with several important regulators of the antioxidant response that are rich in thiol and selenol groups. In this regard, the thioredoxin (Trx) and glutathione GSH systems play important roles in maintaining the redox balance in the brain, a tissue that is prone to oxidative stress due to its high-energy demand [59]. Taking it into account, the following sections address the predominant molecules targeted by MeHg in biological systems, as well as the effects of MeHg on the regulation/modulation of the PI3K/Akt signaling pathway and Nrf2 transcription factor

### 2.1. Glutathione (GSH)

GSH is the most abundant low molecular weight thiol compound synthesized in cells, reaching the concentrations of 1–10 mM, and it is the major antioxidant and redox buffer in human cells. In fact, GSH serves as a reducing agent for ROS and other unstable molecules, in the reaction catalyzed by GPx [60]. Several aspects of MeHg-induced neurotoxicity have been ascribed to GSH depletion. MeHg is able to interact with GSH, leading to the formation of an excretable GS–MeHg complex [61]. This interaction decreases the levels of GSH and, consequently, the GSH:GSSG (disulfide-oxidized) ratio, which contributes to the occurrence of oxidative stress [33]. It is worth mentioning that the pKa of GSH is high at physiological pH conditions and little GSH is ionized. However, at physiological pH values, glutathione S-transferase (GST) effectively lowers the pKa of the cysteine thiol of GSH from ~9.3 in solution to 6.5–7.4 at the active site of various GSTs, resulting in formation of the catalytically active nucleophilic thiolate anion (GS^−^) [62,63].

MeHg has been shown to bind GSH, both in animal models and cell culture systems [64,65]. Indeed, several in vitro [66,67] and in vivo [12,68] evidences have shown that MeHg exposure causes GSH depletion. In addition, GSH and enzymes related to its synthesis are known important targets of MeHg-induced developmental neurotoxicity. For example, Stringari et al. have shown that prenatal exposure to MeHg disrupts the postnatal development of the GSH antioxidant system (GSH levels, GPx and glutathione reductase (GR) activities) in mouse brain. Furthermore, the authors demonstrated that the biochemical alterations endured even when mercury tissue levels decreased and became indistinguishable from those noted in pups born to control dams [68].

Epidemiological and animal studies have shown that MeHg causes loss of neuronal cells in specific brain regions including the visual cortex and the cerebellum [28], with massive loss of cerebellar granule cells (CGC) [69]. Interestingly, the increased sensitivity of CGC has been attributed to the relatively low GSH content of these cells, although it is not the only factor recognized [70,71,72]. In this regard, decreased GSH levels have been reported in the cerebral and cerebellar cortices of MeHg-exposed animals whose cortical mercury levels were in the low micromolar range [12,68]. In the mammalian cerebrum and cerebellum, the intracellular GSH concentrations are in the millimolar (mM) range, therefore the generation of ROS induced by MeHg might be related to GSH-independent mechanisms as well, in addition to its direct interaction with GSH [66,73].

GSH is synthesized by the sequential addition of cysteine to glutamate followed by the addition of glycine. In this sense, MeHg selectively inhibits astrocytic uptake systems for cystine and cysteine transport [74,75], compromising GSH synthesis and the CNS redox potential [76]. The MeHg-induced inhibition of cystine transport and astrocytic GSH production would ultimately lead to decreased neuronal GSH levels and increased glutamate toxicity. It is worth mentioning that one of the major mechanisms involved in MeHg-induced neurotoxicity is glutamate dyshomeostasis. Indeed, inhibition of vesicular glutamate uptake, increase in spontaneous release of glutamate from presynaptic terminals, and inhibition of glutamate uptake by astrocytes are crucial phenomena related to MeHg-mediated neurotoxicity [31,77,78].

Furthermore, studies on the toxicological relevance of MeHg–GSH interaction have demonstrated that strategies able to raise GSH levels are protective against MeHg-induced neurotoxicity [79,80]. For instance, increased ROS formation and depleted mono- and disulfide GSH were observed in neuronal, glial, and mixed cultures, and supplementation with exogenous GSH protected against the MeHg-induced neuronal death [81].

### 2.2. Selenoenzymes

Selenium plays a crucial role in antioxidant defense, as one Se atom is absolutely required at the active site of all selenoenzymes, such as GPx and TrxR, in the form of selenocystein [82]. GPx is an antioxidant enzyme that, in the presence of tripeptide GSH, adds two electrons to reduce H_2_O_2_ and lipid peroxides to water and lipid alcohols, respectively, while simultaneously oxidizing GSH to glutathione disulfide. The GPx/GSH system is thought to be a major defense in low-level oxidative stress, and decreased GPx activity or GHS levels may lead to the absence of adequate H_2_O_2_ and lipid peroxides detoxification, which may be converted to OH-radicals and lipid peroxyl radicals, respectively, by transition metals (Fe^2+^) [83]. Thioredoxin (Trx) is essential for maintaining intracellular redox status. The expression of this small (12 kDa) ubiquitous thiol-active protein is induced by ROS and an elevated serum level may indicate a state of oxidative stress. In this regard, TrxR, a NADPH-dependent lipid hydroperoxide reductase, uses NADPH to maintain the levels of reduced Trx via a mechanism similar to that used by GR to maintain GSH levels, contributing to the maintenance of thiol redox homeostasis in proteins. Importantly, the inhibition of TrxR impairs the cyclical regeneration of Trx activity, as Trx remains in the oxidized state [84,85].

The activity of selenoenzymes, such as GPx, may be negatively affected by MeHg [38,41]. In addition, TxrR may be particularly sensitive to mercury compounds after both in vitro [86,87] and in vivo exposure [39,88]. Glutathione peroxidase isoform 1 (GPx1) is an initial molecular target in CGC exposed to nanomolar concentrations of MeHg (300 nM) for 4 days in vitro [37]. Interestingly, the authors have shown that GPx-1 inhibition occurred before any changes on potential targets that are normally affected by high-dose MeHg exposures. Moreover, Gpx1 overexpression was able to prevent MeHg-induced neuronal death in these cells [37], suggesting that selenoproteins may help to prevent oxidative stress induced when MeHg directly disrupts proteins involved in cellular redox system pathways. Of particular toxicological significance, the results obtained by Farina et al. 2009 [37] indicate the potential role of Gpx1 as a primary target of MeHg. In agreement, another study has demonstrated that MeHg decreased GPx activity in three different models: (a) in mouse brain after treatment with MeHg (40 mg/L in drinking water), (b) in mouse brain mitochondrial-enriched fractions isolated from MeHg-treated animals, and (c) in cultured human neuroblastoma SH-SY5Y cells [38]. The authors have concluded that the inhibition of this antioxidant enzyme may cause a significant increase of MeHg-induced impairment of cell viability and oxidative stress. Ruszkiewicz et al., 2016 have demonstrated that MeHg affects the antioxidant Trx and GSH systems in a sex-and structure-specific manner, and that these changes are not associated with altered mRNA expression, but rather posttranscriptional mechanisms [89]. Although the direct interaction of MeHg with the selenol group of the selenoproteins is an important molecular mechanism involved in the MeHg-induced cytotoxicity and decreased protein function [37,90], posttranscriptional events concerning mercury–selenium interaction seem to be also involved in the decreased activity of these selenoproteins [91,92].

Se deficiency has been reported to exacerbate the neurodevelopmental effects induced by MeHg [93,94]. In fact, maternal exposure to MeHg decreased Se concentration and impaired GPx activity in the neural tissue of the offspring, but not in the maternal neural tissue. In addition, MeHg exposure of Se-deficient perinatal mice resulted in retarded neurobehavioral development [94]. A relatively recent study corroborates the hypothesis that Se deficiency is a contributing factor to MeHg-induced toxicity. The authors have shown that selenoprotein genes from antioxidant pathways, such as members of the GPx and TrxR families, were exclusively downregulated by MeHg in whole zebrafish (Danio rerio) embryos, and then rescued by elevated Se levels [91]. These results suggest that Se can reduce the toxicity of MeHg, although the mechanisms behind this interaction have yet to be fully clarified. In addition, it has been shown that the selenocompounds diphenyl diselenide (PhSe)_2_ and ebselen possess thiol peroxidase-like activity, and neuroprotective properties against MeHg-induced neurotoxicity in vivo and in vitro [95,96,97,98]. Using an in vitro experimental model of cultured human neuroblastoma cells (SH-SY5Y), a recent study has shown that MeHg was able to inhibit the activity of Gpx and TrxR, while coexposure to (PhSe)_2_ and MeHg showed a protective effect on both the activity and expression of TrxR [99]. The authors have speculated that a direct interaction of MeHg with the selenol groups of these enzymes and/or a reduction in enzyme synthesis may possibly be related to the inhibition of TrxR and GPx activities [33].

## 3. MeHg and PI3K/Akt Signaling Pathway

The phosphoinositide-3 kinase (PI3K)/Akt signaling pathway has been extensively reviewed by several authors [100,101,102]. Briefly, activation of receptor tyrosine kinases (RTK) or G-protein-coupled receptors (GPCR) lead the recruitment of PI3K in the plasma membrane. Following its recruitment, PI3K is activated and phosphorylates phosphatidylinositol (4,5) biphosphate (PIP_2_) to phosphatidylinositol (3,4 5)-triphosphate (PIP_3_). PIP_3_ recruits Akt to the plasmatic membrane, allowing its activation through phosphorylation on threonine 308 and serine 473 by phospho-inositide-dependent kinase-1 (PDK1) [103] and mammalian target of rapamycin complex 2 (mTORC2) [104], respectively. In addition, negative regulation of the pathway is mediated by phosphatase and tensin homolog (PTEN), which dephosphorylates PIP_3_ to PIP_2_ [105]. It is well known that the PI3K/Akt signaling pathway plays a key role in multiple cellular processes, including metabolism, cell survival, proliferation, and motility [102], to name a few. In this regard, the activation of the PI3K/Akt signaling pathway can culminate in the inhibition of apoptosis in several cellular models [106,107,108], whereas the inhibition of this pathway could be associated with decreased cell viability [109]. Furthermore, it has been shown that ROS can activate the PI3K/Akt signaling pathway and inhibit PTEN [110,111], thus, inducing the Akt activation, as shown in several cultured cell models, such as fibroblasts [112], vascular smooth muscle cells [113], and mesanglial cells [114]. Sonoda et al., 1999 have demonstrated that the activation of Akt by hydrogen peroxide (H_2_O_2_) in a glioblastoma cell line was blocked by wortmannin, a PI3K inhibitor, culminating in increased cell death [115].

Moreover, MeHg can also modulate the PI3K/Akt signaling pathway. Indeed, exposure to 100 nM MeHg for 24 h induced apoptosis in differentiating PC12 cells, and decreased Akt phosphorylation [116]. Corroborating these findings, Pierozan et al., 2017 have also demonstrated that MeHg reduced neuronal viability and induced caspase 3-dependent apoptosis with downregulated PI3K/Akt pathway in primary cortical neurons treated with 1 µM MeHg for 24 h. In this study, the authors noted increased oxidative damage, suggesting that MeHg downregulated Akt phosphorylation despite the noted increase in ROS production [117]. A recent in vivo study using 30-day-old pups from pregnant rats treated with 2 mg/kg MeHg from gestational day 5 until parturition, has shown that the pups prenatally exposed to MeHg failed to increase hippocampal oxidative stress, while the levels of Akt phosphorylation were decreased. These results suggest that MeHg may regulate the Akt signaling pathway by a mechanism independent of ROS generation [9].

On the other hand, a submicromolar MeHg exposure (1 µmol/L) induced Akt activation, probably secondary to increased ROS production (in pancreatic β cell—derived HIT-T15 cells). Moreover, the antioxidant *N*-acetyl-l-cysteine (NAC) prevented MeHg-induced upregulation of Akt phosphorylation but did not reverse PI3K activity in these cells [118]. Similar results were obtained in isolated mouse pancreatic islets [118], suggesting that MeHg-induced oxidative stress may be involved in the regulation of Akt phosphorylation independently of PI3K in pancreatic cells. The same authors have shown similar results in mice treated for 2–4 weeks with 20 µg/kg MeHg, demonstrating an increase in oxidative stress parameters and upregulation of Akt phosphorylation in pancreatic islets [118]. Furthermore, a low-dose MeHg exposure (up to 2 µM) increased Akt phosphorylation, presumably by inactivation of PTEN through S-mercuration (in neuroblastoma SH-SY5Y cell line). Pretreatment with the PI3K/Akt inhibitor, wortmannin, enhanced MeHg-induced cytotoxicity [119]. In this study, the authors have suggested that low-dose MeHg exposures might be associated with activation of cell survival responses [120].

## 4. MeHg Regulation of Nrf2 Activity

As mentioned before, the increase in ROS generation and the disruption of the antioxidant defense system are primary mechanisms related to MeHg-induced cell toxicity and neurodegeneration [1,33]. In this sense, a recent body of evidence has suggested that MeHg can modulate the Nrf2, the transcription factor involved in the regulation of the antioxidant defense system. Nrf2 is a transcription factor that belongs to cap ‘n’ collar (CNC) family, with a basic leucine zipper in its structure. This protein is an important regulator of the antioxidant cell response, regulating the expression of around 1055 genes involved not only in the antioxidant defenses, but also in cell proliferation, metabolism, and immune and detoxifying responses. Once activated, Nrf2 is translocated to the cell nucleus and forms heterodimers with other transcript such as c-Jun and small Maf proteins, binding to the antioxidant response element (ARE: 5′-GTGACNNNGC-3′), favoring the transcription of Nfr2-related genes [121,122,123]. The main regulator of Nrf2 activity is the Kelch-like ECH-associated protein 1 (Keap1) protein [123]. In basal conditions, Keap-1 is associated to Nrf2 inducing Nrf2 ubiquitination by E3 ubiquitin ligase, and consequent degradation through 26S proteasome. The Keap-1 structure contain some reactive cysteine residues (Cys151, Cys273, Cys288 and Cys297), which can be modified under oxidative stress conditions or by electrophilic compounds, promoting the disruption of the Keap-1/Nrf2 association and Nrf2 translocation to the cell nucleus, and consequent gene expression [121,123,124]. In addition, besides the negative regulation of Keap-1 on Nrf2 activity, the modulation of this protein by other signaling pathways has also been described. In fact, nuclear translocation of Nrf2 may involve the PKC and AMPK-dependent phosphorylation [125,126]. An association between PI3K/Akt activation and Nrf2 up-regulation has also been reported [127,128,129,130]. Once activated, Akt promotes the inhibition of GSK-3β through phosphorylation on ser-9. GSK-3β can induce Nrf2 phosphorylation, creating a degradation domain that is recognized by the ubiquitin ligase, targeting Nrf2 for proteasomal degradation [131,132,133,134]. GSK-3β can also phosphorylate a member of the src-kinase family named Fyn, which can phosphorylate Nrf2, favoring its export from the cell nucleus [130,135].

Since MeHg is a potent oxidative stress inducer and an electrophilic agent, the activation and up-regulation of Nrf2 have been described upon exposure to this metal. This hypothesis is supported by numerous data showing an increase in the Nrf2-related gene expression (such as: ho-1, nqo-1, cglc and Nrf2), and also an increase in the Nrf2 nuclear translocation, after exposures to MeHg in vitro [67,71,120,136] and in vivo [137]. Furthermore, it has been demonstrated that Nrf2 genetic knockdown can increase the susceptibility to MeHg toxicity, and treatment with compounds that are able to increase Nrf2 activity ameliorate some consequences of MeHg-induced toxicity [136,138]. In this sense, it seems that MeHg-induced Nrf2 activation is associated with a cellular defense mechanism in response to this metal exposure.

MeHg is able to disrupt the Nrf2/Keap-1 association, which may be associated with an increase in the Nrf2 activation [136,139]. It has been shown a direct interaction between MeHg and the cysteine residues in Keap-1 structure (Cys151, Cys368, and Cys489) [136,139,140]. As mentioned previously, MeHg can react with GSH to form a MeHg-SG adduct that is transferred to extracellular space by the multidrug-resistance associated protein (MRP) [1]. This GSH adduct readily undergoes S-transmercuration with cellular proteins, inducing the mercuration of Cys319 residue in the Keap-1 structure [141]. Cys151 residue is essential to electrophile-mediated disassociation of Keap1 from Nrf2, and The Cys319 residue is critical to the ubiquitin E3 ligase activity and consequent Nrf2 degradation [136,142,143]. In this sense, it is possible to speculate that the MeHg-induced modifications in these cysteine residues, through direct interaction with Cys151 and Cys319 or by ROS-mediated modifications, could disrupt the inhibitory effect of Keap-1 in Nrf2 activation, allowing its translocation to the cell nucleus. It is noteworthy that the cysteine residues in Keap-1 are also potential targets for oxidation mediated by ROS [124,130]. However, further studies are necessary to elucidate the role of ROS in MeHg-induced Nrf2 activation.

MeHg can induce the modulation of PI3k/Akt, and the inhibition of this signaling pathway was able to attenuate the MeHg-induced Nrf2 activation [71]. An increase in GSK-3β phosphorylation was also observed 6 h after an in vitro MeHg exposure in primary cortical astrocytes. MeHg decreased the expression of Fyn and Sp1 (the transcription factor associated to fyn expression), and Fyn nuclear localization, which may possibly suggest a Fyn downregulation in these cells [120]. Thus, it is speculated that MeHg promotes Akt activation and consequent inhibitory phosphorylation of GSK-3β. In these conditions, the downregulation of Fyn phosphorylation may inhibit the Fyn-mediated Nrf2 nuclear export, enhancing the Nrf2 nuclear localization. These authors have also shown that the increase in the Nrf2-related genes was followed by downregulation of Sp1-related genes (such as *fyn* and *tgf-β1*) [120]. This finding corroborates the idea that Sp1 can interact with Nrf2 at promoter sequences, suppressing the expression of Sp1 specific target genes [144].

## 5. Conclusions

MeHg is a hazardous environmental pollutant of great concern to public health because of its neurotoxic effects. Due to its electrophilic nature, MeHg can react with nucleophiles such as sulfhydryl- and selenol-containing proteins and low-molecular-weight molecules in biological systems. MeHg’s affinity for the anionic form of thiol and selenol groups is extremely high and responsible for most of its toxicological effects. Indeed, MeHg can interfere with several crucial regulators of the antioxidant response that are rich in thiol and selenol groups such as glutathione (GSH), the major antioxidant and redox buffer in human cells, and the antioxidant selenoenzymes thioredoxin reductase (TrxR) and glutathione peroxidase (Gpx). Consequently, the excessive generation of ROS is an important phenomena that culminates in cell death. In this regard, antioxidant molecules have been reported as important protective agents against MeHg-induced toxicity. Our understanding of the critical targets of MeHg is incomplete, and detailed experimental data regarding the mechanism of action are needed. For instance, given that MeHg is known to generate ROS, and that mammalian cells activate Nrf2-mediated transcription in response to ROS, it is not surprising that Nrf2 activation has been demonstrated in response to MeHg exposure. However, relatively little information has been found about the potential mechanisms involved in MeHg-induced Nrf2 activation. In addition, the relationship between the PI3K/Akt signaling pathway and MeHg toxicity is still very limited, and consideration should be given to future research. In conclusion, the data presented in this review suggest that multiple mechanisms are involved in MeHg-induced oxidative stress and cell toxicity. Taken together, the data highlight several promising directions for future research in this area.

## References

[1] Farina M., Aschner M., Rocha J.B. (2011). Oxidative stress in MeHg-induced neurotoxicity. Toxicol. Appl. Pharmacol..

[2] Compeau G.C., Bartha R. (1985). Sulfate-reducing bacteria: Principal methylators of mercury in anoxic estuarine sediment. Appl. Environ. Microb..

[3] Hintelmann H. (2010). Organomercurials. Their formation and pathways in the environment. Met. Ions Life Sci..

[4] Clarkson T.W., Magos L., Myers G.J. (2003). The toxicology of mercury—Current exposures and clinical manifestations. New Engl. J. Med..

[5] Renieri E.A., Alegakis A.K., Kiriakakis M., Vinceti M., Ozcagli E., Wilks M.F., Tsatsakis A.M. (2014). Cd, Pb and Hg Biomonitoring in Fish of the Mediterranean Region and Risk Estimations on Fish Consumption. Toxics.

[6] Zareba G., Cernichiari E., Hojo R., Nitt S.M., Weiss B., Mumtaz M.M., Jones D.E., Clarkson T.W. (2007). Thimerosal distribution and metabolism in neonatal mice: Comparison with methyl mercury. J. Appl. Toxicol..

[7] Costa L.G., Aschner M., Vitalone A., Syversen T., Soldin O.P. (2004). Developmental neuropathology of environmental agents. Annu. Rev. Pharmacol. Toxicol..

[8] Hassan S.A., Moussa E.A., Abbott L.C. (2012). The effect of methylmercury exposure on early central nervous system development in the zebrafish (Danio rerio) embryo. J. Appl. Toxicol. JAT.

[9] Heimfarth L., Delgado J., Mignori M.R., Gelain D.P., Moreira J.C.F., Pessoa-Pureur R. (2018). Developmental neurotoxicity of the hippocampus following in utero exposure to methylmercury: Impairment in cell signaling. Arch. Toxicol..

[10] Johansson C., Castoldi A.F., Onishchenko N., Manzo L., Vahter M., Ceccatelli S. (2007). Neurobehavioural and molecular changes induced by methylmercury exposure during development. Neurotox. Res..

[11] Marsh D.O., Clarkson T.W., Myers G.J., Davidson P.W., Cox C., Cernichiari E., Tanner M.A., Lednar W., Shamlaye C., Choisy O. (1995). The seychelles study of fetal methylmercury exposure and child development: Introduction. Neurotoxicology.

[12] Franco J.L., Teixeira A., Meotti F.C., Ribas C.M., Stringari J., Garcia Pomblum S.C., Moro A.M., Bohrer D., Bairros A.V., Dafre A.L. (2006). Cerebellar thiol status and motor deficit after lactational exposure to methylmercury. Environ. Res..

[13] Manfroi C.B., Schwalm F.D., Cereser V., Abreu F., Oliveira A., Bizarro L., Rocha J.B., Frizzo M.E., Souza D.O., Farina M. (2004). Maternal milk as methylmercury source for suckling mice: Neurotoxic effects involved with the cerebellar glutamatergic system. Toxicol. Sci..

[14] Debes F., Budtz-Jorgensen E., Weihe P., White R.F., Grandjean P. (2006). Impact of prenatal methylmercury exposure on neurobehavioral function at age 14 years. Neurotoxicol. Teratol..

[15] Grandjean P., Weihe P., White R.F., Debes F., Araki S., Yokoyama K., Murata K., Sorensen N., Dahl R., Jorgensen P.J. (1997). Cognitive deficit in 7-year-old children with prenatal exposure to methylmercury. Neurotoxicol. Teratol..

[16] Tatsuta N., Murata K., Iwai-Shimada M., Yaginuma-Sakurai K., Satoh H., Nakai K. (2017). Psychomotor ability in children prenatally exposed to methylmercury: The 18-month follow-up of Tohoku study of child development. Tohoku J. Exp. Med..

[17] Choi B.H. (1986). Methylmercury poisoning of the developing nervous system: I. Pattern of neuronal migration in the cerebral cortex. Neurotoxicology.

[18] Choi B.H., Lapham L.W., Amin-Zaki L., Saleem T. (1978). Abnormal neuronal migration, deranged cerebral cortical organization, and diffuse white matter astrocytosis of human fetal brain: A major effect of methylmercury poisoning in utero. J. Neuropathol. Exp. Neurol..

[19] Bjorklund O., Kahlstrom J., Salmi P., Ogren S.O., Vahter M., Chen J.F., Fredholm B.B., Dare E. (2007). The effects of methylmercury on motor activity are sex- and age-dependent, and modulated by genetic deletion of adenosine receptors and caffeine administration. Toxicology.

[20] Carratu M.R., Borracci P., Coluccia A., Giustino A., Renna G., Tomasini M.C., Raisi E., Antonelli T., Cuomo V., Mazzoni E. (2006). Acute exposure to methylmercury at two developmental windows: Focus on neurobehavioral and neurochemical effects in rat offspring. Neuroscience.

[21] Dare E., Fetissov S., Hokfelt T., Hall H., Ogren S.O., Ceccatelli S. (2003). Effects of prenatal exposure to methylmercury on dopamine-mediated locomotor activity and dopamine D2 receptor binding. Naunyn Schmiedebergs Arch. Pharmacol..

[22] Sakamoto M., Kakita A., Wakabayashi K., Takahashi H., Nakano A., Akagi H. (2002). Evaluation of changes in methylmercury accumulation in the developing rat brain and its effects: A study with consecutive and moderate dose exposure throughout gestation and lactation periods. Brain Res..

[23] Paletz E.M., Craig-Schmidt M.C., Newland M.C. (2006). Gestational exposure to methylmercury and n-3 fatty acids: Effects on high- and low-rate operant behavior in adulthood. Neurotoxicol. Teratol..

[24] Sfakianakis D.G., Renieri E., Kentouri M., Tsatsakis A.M. (2015). Effect of heavy metals on fish larvae deformities: A review. Environ. Res..

[25] Carvalho M.C., Franco J.L., Ghizoni H., Kobus K., Nazari E.M., Rocha J.B., Nogueira C.W., Dafre A.L., Muller Y.M., Farina M. (2007). Effects of 2,3-dimercapto-1-propanesulfonic acid (DMPS) on methylmercury-induced locomotor deficits and cerebellar toxicity in mice. Toxicology.

[26] Farina M., Franco J.L., Ribas C.M., Meotti F.C., Missau F.C., Pizzolatti M.G., Dafre A.L., Santos A.R. (2005). Protective effects of polygala paniculata extract against methylmercury-induced neurotoxicity in mice. J. Pharm. Pharmacol..

[27] Zimmermann L.T., dos Santos D.B., Colle D., dos Santos A.A., Hort M.A., Garcia S.C., Bressan L.P., Bohrer D., Farina M. (2014). Methionine stimulates motor impairment and cerebellar mercury deposition in methylmercury-exposed mice. J. Toxicol. Environ. Health A.

[28] Aschner M., Syversen T. (2005). Methylmercury: Recent advances in the understanding of its neurotoxicity. Ther. Drug Monit..

[29] Sakamoto M., Nakano A., Kajiwara Y., Naruse I., Fujisaki T. (1993). Effects of methyl mercury in postnatal developing rats. Environ. Res..

[30] Kaur P., Aschner M., Syversen T. (2006). Glutathione modulation influences methyl mercury induced neurotoxicity in primary cell cultures of neurons and astrocytes. Neurotoxicology.

[31] Aschner M., Syversen T., Souza D.O., Rocha J.B., Farina M. (2007). Involvement of glutamate and reactive oxygen species in methylmercury neurotoxicity. Braz. J. Med. Biol. Res..

[32] Ceccatelli S., Dare E., Moors M. (2010). Methylmercury-induced neurotoxicity and apoptosis. Chem. Biol. Interact..

[33] Farina M., Rocha J.B., Aschner M. (2011). Mechanisms of methylmercury-induced neurotoxicity: Evidence from experimental studies. Life Sci..

[34] Choi D.W. (1992). Excitotoxic cell death. J. Neurobiol..

[35] Lafon-Cazal M., Pietri S., Culcasi M., Bockaert J. (1993). Nmda-dependent superoxide production and neurotoxicity. Nature.

[36] Park S.T., Lim K.T., Chung Y.T., Kim S.U. (1996). Methylmercury-induced neurotoxicity in cerebral neuron culture is blocked by antioxidants and NMDA receptor antagonists. Neurotoxicology.

[37] Farina M., Campos F., Vendrell I., Berenguer J., Barzi M., Pons S., Sunol C. (2009). Probucol increases glutathione peroxidase-1 activity and displays long-lasting protection against methylmercury toxicity in cerebellar granule cells. Toxicol. Sci..

[38] Franco J.L., Posser T., Dunkley P.R., Dickson P.W., Mattos J.J., Martins R., Bainy A.C., Marques M.R., Dafre A.L., Farina M. (2009). Methylmercury neurotoxicity is associated with inhibition of the antioxidant enzyme glutathione peroxidase. Free Radic. Biol. Med..

[39] Wagner C., Sudati J.H., Nogueira C.W., Rocha J.B. (2010). In vivo and in vitro inhibition of mice thioredoxin reductase by methylmercury. Biometals.

[40] Yin Z., Milatovic D., Aschner J.L., Syversen T., Rocha J.B., Souza D.O., Sidoryk M., Albrecht J., Aschner M. (2007). Methylmercury induces oxidative injury, alterations in permeability and glutamine transport in cultured astrocytes. Brain Res..

[41] Glaser V., Nazari E.M., Muller Y.M., Feksa L., Wannmacher C.M., Rocha J.B., de Bem A.F., Farina M., Latini A. (2010). Effects of inorganic selenium administration in methylmercury-induced neurotoxicity in mouse cerebral cortex. Int. J. Dev. Neurosci..

[42] Osburn W.O., Kensler T.W. (2008). Nrf2 signaling: An adaptive response pathway for protection against environmental toxic insults. Mutat. Res..

[43] Nakaso K., Yano H., Fukuhara Y., Takeshima T., Wada-Isoe K., Nakashima K. (2003). Pi3k is a key molecule in the Nrf2-mediated regulation of antioxidative proteins by hemin in human neuroblastoma cells. FEBS Lett..

[44] Wang L., Chen Y., Sternberg P., Cai J. (2008). Essential roles of the Pi3 kinase/Akt pathway in regulating Nrf2-dependent antioxidant functions in the RPE. Investig. Ophthalmol. Vis. Sci..

[45] Hughes W.L. (1957). A physicochemical rationale for the biological activity of mercury and its compounds. Ann. N. Y. Acad. Sci..

[46] Naganuma A., Imura N. (1979). Methylmercury binds to a low molecular weight substance in rabbit and human erythrocytes. Toxicol. Appl. Pharmacol..

[47] Rabenstein D.L., Fairhurst M.T. (1975). Nuclear magnetic resonance studies of the solution chemistry of metal complexes. XI. The binding of methylmercury by sulfhydryl-containing amino acids and by glutathione. J. Am. Chem. Soc..

[48] Thomas D.J., Smith J.C. (1982). Effects of coadministered low-molecular-weight thiol compounds on short-term distribution of methyl mercury in the rat. Toxicol. Appl. Pharmacol..

[49] Omata S., Sakimura K., Ishii T., Sugano H. (1978). Chemical nature of a methylmercury complex with a low molecular weight in the liver cytosol of rats exposed to methylmercury chloride. Biochem. Pharmacol..

[50] Refsvik T., Norseth T. (1975). Methyl mercuric compounds in rat bile. Acta Pharmacol. Toxicol..

[51] Ballatori N., Clarkson T.W. (1985). Biliary secretion of glutathione and of glutathione-metal complexes. Fundam. Appl. Toxicol..

[52] Aschner M., Clarkson T.W. (1988). Uptake of methylmercury in the rat brain: Effects of amino acids. Brain Res..

[53] Yin Z., Jiang H., Syversen T., Rocha J.B., Farina M., Aschner M. (2008). The methylmercury-L-cysteine conjugate is a substrate for the l-type large neutral amino acid transporter. J. Neurochem..

[54] Holben D.H., Smith A.M. (1999). The diverse role of selenium within selenoproteins: A review. J. Am. Diet. Assoc..

[55] Papp L.V., Lu J., Holmgren A., Khanna K.K. (2007). From selenium to selenoproteins: Synthesis, identity, and their role in human health. Antioxid. Redox Signal..

[56] Steinbrenner H., Sies H. (2009). Protection against reactive oxygen species by selenoproteins. Biochim. Biophys. Acta.

[57] Lu J., Holmgren A. (2009). Selenoproteins. J. Biol. Chem..

[58] Sugiura Y., Tamai Y., Tanaka H. (1978). Selenium protection against mercury toxicity: High binding affinity of methylmercury by selenium-containing ligands in comparison with sulfur-containing ligands. Bioinorg. Chem..

[59] Ren X., Zou L., Zhang X., Branco V., Wang J., Carvalho C., Holmgren A., Lu J. (2017). Redox signaling mediated by thioredoxin and glutathione systems in the central nervous system. Antioxid. Redox Signal..

[60] Forman H.J., Zhang H., Rinna A. (2009). Glutathione: Overview of its protective roles, measurement, and biosynthesis. Mol. Aspects Med..

[61] Ballatori N., Clarkson T.W. (1982). Developmental changes in the biliary excretion of methylmercury and glutathione. Science.

[62] Graminski G.F., Kubo Y., Armstrong R.N. (1989). Spectroscopic and kinetic evidence for the thiolate anion of glutathione at the active site of glutathione S-transferase. Biochemistry.

[63] Cheng H., Tchaikovskaya T., Tu Y.S., Chapman J., Qian B., Ching W.M., Tien M., Rowe J.D., Patskovsky Y.V., Listowsky I. (2001). Rat glutathione s-transferase M4-4: An isoenzyme with unique structural features including a redox-reactive cysteine-115 residue that forms mixed disulphides with glutathione. Biochem. J..

[64] Khan H., Khan M.F., Jan S.U., Mukhtiar M., Ullah N., Anwar N. (2012). Role of glutathione in protection against mercury induced poisoning. Pak. J. Pharm. Sci..

[65] Patrick L. (2002). Mercury toxicity and antioxidants: Part 1: Role of glutathione and alpha-lipoic acid in the treatment of mercury toxicity. Altern. Med. Rev..

[66] Franco J.L., Braga H.C., Stringari J., Missau F.C., Posser T., Mendes B.G., Leal R.B., Santos A.R., Dafre A.L., Pizzolatti M.G. (2007). Mercurial-induced hydrogen peroxide generation in mouse brain mitochondria: Protective effects of quercetin. Chem. Res. Toxicol..

[67] Ni M., Li X., Yin Z., Sidoryk-Wegrzynowicz M., Jiang H., Farina M., Rocha J.B., Syversen T., Aschner M. (2011). Comparative study on the response of rat primary astrocytes and microglia to methylmercury toxicity. Glia.

[68] Stringari J., Nunes A.K., Franco J.L., Bohrer D., Garcia S.C., Dafre A.L., Milatovic D., Souza D.O., Rocha J.B., Aschner M. (2008). Prenatal methylmercury exposure hampers glutathione antioxidant system ontogenesis and causes long-lasting oxidative stress in the mouse brain. Toxicol. Appl. Pharmacol..

[69] Takeuchi T. (1982). Pathology of minamata disease. With special reference to its pathogenesis. Acta Pathol. Jpn..

[70] Kaur P., Aschner M., Syversen T. (2007). Role of glutathione in determining the differential sensitivity between the cortical and cerebellar regions towards mercury-induced oxidative stress. Toxicology.

[71] Wang L., Jiang H., Yin Z., Aschner M., Cai J. (2009). Methylmercury toxicity and Nrf2-dependent detoxification in astrocytes. Toxicol. Sci..

[72] Yee S., Choi B.H. (1996). Oxidative stress in neurotoxic effects of methylmercury poisoning. Neurotoxicology.

[73] Mori K., Yoshida K., Nakagawa Y., Hoshikawa S., Ozaki H., Ito S., Watanabe C. (2007). Methylmercury inhibition of type II 5′-deiodinase activity resulting in a decrease in growth hormone production in gh3 cells. Toxicology.

[74] Allen J.W., Shanker G., Aschner M. (2001). Methylmercury inhibits the in vitro uptake of the glutathione precursor, cystine, in astrocytes, but not in neurons. Brain Res..

[75] Shanker G., Allen J.W., Mutkus L.A., Aschner M. (2001). Methylmercury inhibits cysteine uptake in cultured primary astrocytes, but not in neurons. Brain Res..

[76] Aschner M., Yao C.P., Allen J.W., Tan K.H. (2000). Methylmercury alters glutamate transport in astrocytes. Neurochem. Int..

[77] Shanker G., Aschner M. (2001). Identification and characterization of uptake systems for cystine and cysteine in cultured astrocytes and neurons: Evidence for methylmercury-targeted disruption of astrocyte transport. J. Neurosci. Res..

[78] Shanker G., Aschner M. (2003). Methylmercury-induced reactive oxygen species formation in neonatal cerebral astrocytic cultures is attenuated by antioxidants. Brain Res. Mol. Brain Res..

[79] Kaur P., Aschner M., Syversen T. (2011). Biochemical factors modulating cellular neurotoxicity of methylmercury. J. Toxicol..

[80] Shanker G., Syversen T., Aschner J.L., Aschner M. (2005). Modulatory effect of glutathione status and antioxidants on methylmercury-induced free radical formation in primary cultures of cerebral astrocytes. Brain Res. Mol. Brain Res..

[81] Rush T., Liu X., Nowakowski A.B., Petering D.H., Lobner D. (2012). Glutathione-mediated neuroprotection against methylmercury neurotoxicity in cortical culture is dependent on mrp1. Neurotoxicology.

[82] Rayman M.P. (2000). The importance of selenium to human health. Lancet.

[83] Brigelius-Flohe R., Maiorino M. (2013). Glutathione peroxidases. Biochim. Biophys. Acta.

[84] Bjornstedt M., Hamberg M., Kumar S., Xue J., Holmgren A. (1995). Human thioredoxin reductase directly reduces lipid hydroperoxides by nadph and selenocystine strongly stimulates the reaction via catalytically generated selenols. J. Biol. Chem..

[85] Zhong L., Holmgren A. (2002). Mammalian thioredoxin reductases as hydroperoxide reductases. Methods Enzymol..

[86] Carvalho C.M., Chew E.H., Hashemy S.I., Lu J., Holmgren A. (2008). Inhibition of the human thioredoxin system. A molecular mechanism of mercury toxicity. J. Biol. Chem..

[87] Carvalho C.M., Lu J., Zhang X., Arner E.S., Holmgren A. (2011). Effects of selenite and chelating agents on mammalian thioredoxin reductase inhibited by mercury: Implications for treatment of mercury poisoning. FASEB J..

[88] Dalla Corte C.L., Wagner C., Sudati J.H., Comparsi B., Leite G.O., Busanello A., Soares F.A., Aschner M., Rocha J.B. (2013). Effects of diphenyl diselenide on methylmercury toxicity in rats. Biomed. Res. Int..

[89] Ruszkiewicz J.A., Bowman A.B., Farina M., Rocha J.B.T., Aschner M. (2016). Sex- and structure-specific differences in antioxidant responses to methylmercury during early development. Neurotoxicology.

[90] Branco V., Canario J., Lu J., Holmgren A., Carvalho C. (2012). Mercury and selenium interaction in vivo: Effects on thioredoxin reductase and glutathione peroxidase. Free Radic. Biol. Med..

[91] Penglase S., Hamre K., Ellingsen S. (2014). Selenium prevents downregulation of antioxidant selenoprotein genes by methylmercury. Free Radic. Biol. Med..

[92] Usuki F., Yamashita A., Fujimura M. (2011). Post-transcriptional defects of antioxidant selenoenzymes cause oxidative stress under methylmercury exposure. J. Biol. Chem..

[93] Fredriksson A., Gardlund A.T., Bergman K., Oskarsson A., Ohlin B., Danielsson B., Archer T. (1993). Effects of maternal dietary supplementation with selenite on the postnatal development of rat offspring exposed to methyl mercury in utero. Pharmacol. Toxicol..

[94] Watanabe C., Yin K., Kasanuma Y., Satoh H. (1999). In utero exposure to methylmercury and Se deficiency converge on the neurobehavioral outcome in mice. Neurotoxicol. Teratol..

[95] de Freitas A.S., Funck V.R., Rotta Mdos S., Bohrer D., Morschbacher V., Puntel R.L., Nogueira C.W., Farina M., Aschner M., Rocha J.B. (2009). Diphenyl diselenide, a simple organoselenium compound, decreases methylmercury-induced cerebral, hepatic and renal oxidative stress and mercury deposition in adult mice. Brain Res. Bull..

[96] Farina M., Dahm K.C., Schwalm F.D., Brusque A.M., Frizzo M.E., Zeni G., Souza D.O., Rocha J.B. (2003). Methylmercury increases glutamate release from brain synaptosomes and glutamate uptake by cortical slices from suckling rat pups: Modulatory effect of ebselen. Toxicol. Sci..

[97] Farina M., Frizzo M.E., Soares F.A., Schwalm F.D., Dietrich M.O., Zeni G., Rocha J.B., Souza D.O. (2003). Ebselen protects against methylmercury-induced inhibition of glutamate uptake by cortical slices from adult mice. Toxicol. Lett..

[98] Roos D.H., Puntel R.L., Santos M.M., Souza D.O., Farina M., Nogueira C.W., Aschner M., Burger M.E., Barbosa N.B., Rocha J.B. (2009). Guanosine and synthetic organoselenium compounds modulate methylmercury-induced oxidative stress in rat brain cortical slices: Involvement of oxidative stress and glutamatergic system. Toxicol. In Vitro.

[99] Meinerz D.F., Branco V., Aschner M., Carvalho C., Rocha J.B.T. (2017). Diphenyl diselenide protects against methylmercury-induced inhibition of thioredoxin reductase and glutathione peroxidase in human neuroblastoma cells: A comparison with ebselen. J. Appl. Toxicol..

[100] Cantley L.C. (2002). The phosphoinositide 3-kinase pathway. Science.

[101] Neri L.M., Borgatti P., Capitani S., Martelli A.M. (2002). The nuclear phosphoinositide 3-kinase/Akt pathway: A new second messenger system. Biochem. Biophys. Acta Biomembr..

[102] Manning B.D., Toker A. (2017). AKT/PKB signaling: Navigating the network. Cell.

[103] Alessi D.R., James S.R., Downes C.P., Holmes A.B., Gaffney P.R., Reese C.B., Cohen P. (1997). Characterization of a 3-phosphoinositide-dependent protein kinase which phosphorylates and activates protein kinase balpha. Curr. Biol..

[104] Sarbassov D.D., Guertin D.A., Ali S.M., Sabatini D.M. (2005). Phosphorylation and regulation of Akt/PKB by the rictor-mtor complex. Science.

[105] Stambolic V., Suzuki A., de la Pompa J.L., Brothers G.M., Mirtsos C., Sasaki T., Ruland J., Penninger J.M., Siderovski D.P., Mak T.W. (1998). Negative regulation of PKB/Akt-dependent cell survival by the tumor suppressor PTEN. Cell.

[106] Das J., Ghosh J., Manna P., Sil P.C. (2011). Taurine suppresses doxorubicin-triggered oxidative stress and cardiac apoptosis in rat via up-regulation of PI3-K/Akt and inhibition of p53, p38-JNK. Biochem. Pharmacol..

[107] Ohashi H., Takagi H., Oh H., Suzuma K., Suzuma I., Miyamoto N., Uemura A., Watanabe D., Murakami T., Sugaya T. (2004). Phosphatidylinositol 3-kinase/Akt regulates angiotensin II-induced inhibition of apoptosis in microvascular endothelial cells by governing survivin expression and suppression of caspase-3 activity. Circ. Res..

[108] Widenmaier S.B., Ao Z., Kim S.J., Warnock G., McIntosh C.H. (2009). Suppression of p38 MAPK and JNK via Akt-mediated inhibition of apoptosis signal-regulating kinase 1 constitutes a core component of the beta-cell pro-survival effects of glucose-dependent insulinotropic polypeptide. J. Biol. Chem..

[109] Hsu A.L., Ching T.T., Wang D.S., Song X., Rangnekar V.M., Chen C.S. (2000). The cyclooxygenase-2 inhibitor celecoxib induces apoptosis by blocking Akt activation in human prostate cancer cells independently of Bcl-2. J. Biol. Chem..

[110] Huang J.S., Cho C.Y., Hong C.C., Yan M.D., Hsieh M.C., Lay J.D., Lai G.M., Cheng A.L., Chuang S.E. (2013). Oxidative stress enhances Axl-mediated cell migration through an Akt1/Rac1-dependent mechanism. Free Radical Bio. Med..

[111] Luo H., Yang Y., Duan J., Wu P., Jiang Q., Xu C. (2013). PTEN-regulated AKT/foxO3a/Bim signaling contributes to reactive oxygen species-mediated apoptosis in selenite-treated colorectal cancer cells. Cell Death Dis..

[112] Esposito F., Chirico G., Montesano Gesualdi N., Posadas I., Ammendola R., Russo T., Cirino G., Cimino F. (2003). Protein kinase b activation by reactive oxygen species is independent of tyrosine kinase receptor phosphorylation and requires SRC activity. J. Biol. Chem..

[113] Ushio-Fukai M., Alexander R.W., Akers M., Yin Q., Fujio Y., Walsh K., Griendling K.K. (1999). Reactive oxygen species mediate the activation of Akt/protein kinase b by angiotensin II in vascular smooth muscle cells. J. Biol. Chem..

[114] Gorin Y., Ricono J.M., Kim N.H., Bhandari B., Choudhury G.G., Abboud H.E. (2003). Nox4 mediates angiotensin ii-induced activation of Akt/protein kinase b in mesangial cells. Am. J. Physiol. Renal.

[115] Sonoda Y., Watanabe S., Matsumoto Y., Aizu-Yokota E., Kasahara T. (1999). Fak is the upstream signal protein of the phosphatidylinositol 3-kinase-Akt survival pathway in hydrogen peroxide-induced apoptosis of a human glioblastoma cell line. J. Biol. Chem..

[116] Fujimura M., Usuki F. (2015). Methylmercury causes neuronal cell death through the suppression of the Trka pathway: In vitro and in vivo effects of Trka pathway activators. Toxicol. Appl. Pharm..

[117] Pierozan P., Biasibetti H., Schmitz F., Avila H., Fernandes C.G., Pessoa-Pureur R., Wyse A.T.S. (2017). Neurotoxicity of methylmercury in isolated astrocytes and neurons: The cytoskeleton as a main target. Mol. Neurobiol..

[118] Chen Y.W., Huang C.F., Tsai K.S., Yang R.S., Yen C.C., Yang C.Y., Lin-Shiau S.Y., Liu S.H. (2006). The role of phosphoinositide 3-kinase/Akt signaling in low-dose mercury-induced mouse pancreatic beta-cell dysfunction in vitro and in vivo. Diabetes.

[119] Unoki T., Abiko Y., Toyama T., Uehara T., Tsuboi K., Nishida M., Kaji T., Kumagai Y. (2016). Methylmercury, an environmental electrophile capable of activation and disruption of the Akt/CREB/Bcl-2 signal transduction pathway in SH-SY5Y cells. Sci. Rep..

[120] Culbreth M., Zhang Z., Aschner M. (2017). Methylmercury augments Nrf2 activity by downregulation of the Src family kinase Fyn. Neurotoxicology.

[121] Silva-Islas C.A., Maldonado P.D. (2018). Canonical and non-canonical mechanisms of Nrf2 activation. Pharmacol. Res..

[122] Kobayashi M., Yamamoto M. (2005). Molecular mechanisms activating the Nrf2-Keap1 pathway of antioxidant gene regulation. Antioxid. Redox Signal..

[123] Canning P., Sorrell F.J., Bullock A.N. (2015). Structural basis of keap1 interactions with Nrf2. Free Radic. Biol. Med..

[124] Kaspar J.W., Niture S.K., Jaiswal A.K. (2009). Nrf2:Inrf2 (Keap1) signaling in oxidative stress. Free Radic. Biol. Med..

[125] Huang H.C., Nguyen T., Pickett C.B. (2002). Phosphorylation of Nrf2 at Ser-40 by protein kinase c regulates antioxidant response element-mediated transcription. J. Biol. Chem..

[126] Joo M.S., Kim W.D., Lee K.Y., Kim J.H., Koo J.H., Kim S.G. (2016). Ampk facilitates nuclear accumulation of Nrf2 by phosphorylating at serine 550. Mol. Cell. Biol..

[127] Calkins M.J., Johnson D.A., Townsend J.A., Vargas M.R., Dowell J.A., Williamson T.P., Kraft A.D., Lee J.M., Li J., Johnson J.A. (2009). The Nrf2/are pathway as a potential therapeutic target in neurodegenerative disease. Antioxid. Redox Signal..

[128] de Oliveira M.R., Ferreira G.C., Schuck P.F., Dal Bosco S.M. (2015). Role for the Pi3K/Akt/Nrf2 signaling pathway in the protective effects of carnosic acid against methylglyoxal-induced neurotoxicity in SH-SY5Y neuroblastoma cells. Chem. Biol. Interact..

[129] Wang L., Zhang S., Cheng H., Lv H., Cheng G., Ci X. (2016). Nrf2-mediated liver protection by esculentoside a against acetaminophen toxicity through the AMPK/Akt/GSK3β pathway. Free Radic. Biol. Med..

[130] Niture S.K., Khatri R., Jaiswal A.K. (2014). Regulation of Nrf2-an update. Free Radic Biol Med.

[131] Cuadrado A. (2015). Structural and functional characterization of Nrf2 degradation by glycogen synthase kinase 3/β-TrCP. Free Radic. Biol. Med..

[132] Rada P., Rojo A.I., Chowdhry S., McMahon M., Hayes J.D., Cuadrado A. (2011). SCF/{beta}-TrCP promotes glycogen synthase kinase 3-dependent degradation of the Nrf2 transcription factor in a keap1-independent manner. Mol. Cell. Biol..

[133] Chowdhry S., Zhang Y., McMahon M., Sutherland C., Cuadrado A., Hayes J.D. (2013). Nrf2 is controlled by two distinct β-TrCP recognition motifs in its Neh6 domain, one of which can be modulated by GSK-3 activity. Oncogene.

[134] Rada P., Rojo A.I., Evrard-Todeschi N., Innamorato N.G., Cotte A., Jaworski T., Tobon-Velasco J.C., Devijver H., Garcia-Mayoral M.F., Van Leuven F. (2012). Structural and functional characterization of Nrf2 degradation by the glycogen synthase kinase 3/beta-trcp axis. Mol. Cell. Biol..

[135] Jain A.K., Jaiswal A.K. (2007). Gsk-3beta acts upstream of Fyn kinase in regulation of nuclear export and degradation of NF-E2 related factor 2. J. Biol. Chem..

[136] Toyama T., Sumi D., Shinkai Y., Yasutake A., Taguchi K., Tong K.I., Yamamoto M., Kumagai Y. (2007). Cytoprotective role of Nrf2/Keap1 system in methylmercury toxicity. Biochem. Biophys. Res. Commun..

[137] Feng S., Xu Z., Wang F., Yang T., Liu W., Deng Y., Xu B. (2017). Sulforaphane prevents methylmercury-induced oxidative damage and excitotoxicity through activation of the Nrf2-ARE pathway. Mol. Neurobiol..

[138] Toyama T., Shinkai Y., Yasutake A., Uchida K., Yamamoto M., Kumagai Y. (2011). Isothiocyanates reduce mercury accumulation via an Nrf2-dependent mechanism during exposure of mice to methylmercury. Environ. Health Perspect..

[139] Toyama T., Shinkai Y., Kaji T., Kumagai Y. (2013). Convenient method to assess chemical modification of protein thiols by electrophilic metals. J. Toxicol. Sci..

[140] Kumagai Y., Kanda H., Shinkai Y., Toyama T. (2013). The role of the Keap1/Nrf2 pathway in the cellular response to methylmercury. Oxid. Med. Cell. Longev..

[141] Yoshida E., Abiko Y., Kumagai Y. (2014). Glutathione adduct of methylmercury activates the Keap1-Nrf2 pathway in SH-SY5Y cells. Chem. Res. Toxicol..

[142] Levonen A.L., Landar A., Ramachandran A., Ceaser E.K., Dickinson D.A., Zanoni G., Morrow J.D., Darley-Usmar V.M. (2004). Cellular mechanisms of redox cell signalling: Role of cysteine modification in controlling antioxidant defences in response to electrophilic lipid oxidation products. Biochem. J..

[143] Taguchi K., Motohashi H., Yamamoto M. (2011). Molecular mechanisms of the Keap1-Nrf2 pathway in stress response and cancer evolution. Genes Cells.

[144] Gao P., Li L., Ji L., Wei Y., Li H., Shang G., Zhao Z., Chen Q., Jiang T., Zhang N. (2014). Nrf2 ameliorates diabetic nephropathy progression by transcriptional repression of TGFβ1 through interactions with c-Jun and SP1. Biochim. Biophys. Acta.

